# Editorial: TRLs (triglycerides-rich lipoproteins): a new target for atherosclerosis

**DOI:** 10.3389/fendo.2024.1540602

**Published:** 2025-01-13

**Authors:** Mei Dong, Yunlong Yang, Kayoko Hosaka, Yun Zhang, Yuxia Zhao

**Affiliations:** ^1^ State Key Laboratory for Innovation and Transformation of Luobing Theory, Key Laboratory of Cardiovascular Remodeling and Function Research, Chinese Ministry of Education, Chinese National Health Commission and Chinese Academy of Medical Sciences, Department of Cardiology, Qilu Hospital of Shandong University, Jinan, China; ^2^ Department of Cellular and Genetic Medicine, School of Basic Medical Sciences, Fudan University, Shanghai, China; ^3^ Department of Microbiology, Tumor and Cell Biology, Karolinska Institute, Stockholm, Sweden; ^4^ Department of Traditional Chinese Medicine, Qilu Hospital, Cheeloo College of Medicine, Shandong University, Jinan, Shandong, China

**Keywords:** triglyceride, lipoprotein, atherosclerosis, apo CIII, lipoprotein lipase (LPL)

This Research Topic highlights triglycerides-rich lipoproteins (TRL), focusing on this new target for atherosclerosis. The diabetic population has a relatively high risk of developing hyperlipidemia, especially TRL dyslipidemia. Accumulating evidence points to the causal role of triglyceride lipoproteins and their cholesterol-enriched remnants in atherogenesis. The pathogenesis and mechanisms involve the ineffective TRL catabolism, the dysfunction of apolipoproteins, lipoprotein lipase (LPL) and its main regulators, such as the Apo CIII, Apo AV, and angiopoietin-like proteins in the partition of TRLs during the fast-fed cycle. According to the pathophysiology and mechanisms of TRL metabolism, the therapeutic strategies might be different in terms of lipolysis, remodeling in the bloodstream, and clearance of remnant particles. Apo CIII and ANGPTL3 are now the main targets due to their roles in regulating LPL activity. However, previous triglyceride-lowering medications, including fibrates, nicotinic acid, and omega-3 PUFAs, had a broad range of effects but limited efficacy. It is urgent to screen new effective therapy for TRL lowering and gold standard method for measuring TRL remnants.

The global prevalence of cardiovascular disease (CVD) continues to steadily increase, making it the leading cause of death worldwide. Atherosclerosis (AS) is the main driving factor of these diseases, which eventually leads to adverse cardiovascular events, seriously affecting the quality of life of patients or leading to death. Triglyceride rich lipoproteins (TRLs) include chylomicrons (CM) and very low-density lipoproteins (VLDL). Cholesterol carried in TRLs, also known as residual cholesterol, is increasingly considered to be an important risk factor for atherosclerosis. In addition, many metabolic diseases, such as type 2 diabetes, metabolic syndrome and nonalcoholic fatty liver disease (NAFLD), have been proved to be related to residual cholesterol. In addition, Choudhury et al. have obtained a positive result that the increase of residual cholesterol value is associated with the high risk of low muscle mass in the Korean population. Bai et al.’s study showed that residual cholesterol is U-shaped correlated with all-cause mortality, and is associated with non cardiovascular, non cerebrovascular, and cancer-related mortality rates. Extensive clinical studies have shown that residual cholesterol is closely related to various diseases such as metabolic disorders and cardiovascular diseases, indicating that TRLs and residual cholesterol may become new and powerful clinical monitoring indicators and risk assessment tools.

With the deepening understanding of the important role of triglycerides in blood, new metabolic markers related to triglycerides have been applied in clinical practice. Based on the (1) research of Liu et al., the ratio of triglycerides to high-density lipoprotein cholesterol (TG/HDL-C) is associated with insulin resistance, high risk of cerebrovascular disease, prognosis of kidney disease, occurrence of coronary heart disease, and prognosis of myocardial infarction patients. In addition, Chen et al. found in their latest study that a higher TG/HDL-C ratio is significantly associated with an increased risk of peripartum cardiomyopathy (PPCM). The plasma atherosclerotic index AIP, calculated as log10 (TG/HDL-C), has been recognized as a new practical marker for assessing cardiac metabolic risks, such as coronary artery disease (CAD) risk and insulin resistance related diseases. However, the relevance of AIP as a prognostic biomarker for coronary artery disease (CAD) remains controversial. Huang et al. demonstrated through a retrospective cohort study that there is a U-shaped relationship between the incidence of repeat target vessel revascularization (TVR) after drug-eluting stent (DES) implantation in AIP and CAD patients, particularly in females. These studies indicate that TG and its cholesterol rich metabolites play important roles in the development of various diseases and may become powerful new clinical biomarkers.

Understanding the metabolic pathways of TG and TRLs and their fundamental pathological mechanisms in AS is crucial. Exogenous TG synthesized in the small intestine and endogenous TG synthesized in the liver are secreted into the bloodstream through CM and VLDL, assembled with apolipoprotein, and then hydrolyzed by lipoprotein lipase (LPL) secreted by adipose tissue storage, muscle tissue, and heart. The residual particles produced during this period are cleared by the liver. TRLs and their residues, which have been retained in the arterial wall for a long time, become proinflammatory after modification, induce monocytes to recruit and differentiate into macrophage phagolipoproteins, and form foam cells. Pro-inflammatory TRL can also induce VSMCs to transform into macrophage like cells, exacerbating pre-existing inflammatory responses by secreting various pro-inflammatory cytokines, thereby promoting atherosclerosis. In addition, other lipoproteins can promote atherosclerosis by regulating the activity of LPL. Elevated ApoCIII levels can inhibit LPL activity and liver uptake of VLDL residues, prolong the retention of TRL residues in plasma, and enhance cholesterol binding. Angiopoietin like protein 3 (ANGPTL3) plays an important role in regulating TRL metabolism by inhibiting LPL activity. This Research Topic includes two key reviews. Gugliucci focuses on the synthesis and catabolism of postprandial chylomicron residues *in vivo*, and proposes important clinical significance and detection methods for postprandial plasma lipoprotein evaluation. Xu et al. investigated the role of TRL residues in promoting AS progression and discussed potential targets for blocking and reducing TRLs. Both reviews emphasize the importance of understanding these processes, which may identify potential new drug targets to reduce or even reverse the occurrence of AS, ultimately improving patient prognosis.

Ain et al. described the genetic and clinical characteristics of patients with lipoprotein lipase deficiency through case analysis and systematic literature review. Familial chylomicronemia (FCS) is a rare recessive genetic disorder, with the majority of FCS cases (approximately 95%) associated with specific pathogenic variations in the lipoprotein lipase (LPL) gene. Research has shown that this LPL deficiency will lead to the accumulation of lipoproteins rich in triglycerides, clinically manifested as hypertriglyceridemia (HTG), as well as a higher risk of pancreatitis and cardiovascular disease. This indicates that LPL may have broad potential in reducing plasma TG and TRLs.

The drugs currently used in clinical practice to reduce triglycerides include beta blockers and niacin, which activate peroxisome proliferator activated receptor alpha (PPAR alpha), induce LPL expression, and reduce serum TG levels (2). In the research meta-analysis of Chukwurah et al., fenofibrate did not significantly improve the ASCVD outcome in individuals with type 2 diabetes, but subgroup analysis showed that these patients had a much lower risk of suffering from the disease. Omega-3 polyunsaturated fatty acid preparations have been tested in large-scale ASCVD outcome studies, and overall, their benefits in reducing ASCVD risk provide only weak evidence. At present, research on creating compounds for treating hypertriglyceridemia mainly focuses on increasing TRL lipolysis. ApoIII and ANGPTL3 are currently the main targets. The current research trend focuses on interfering with or inhibiting ANGPTL3 through various methods, including ANGPTL3 monoclonal antibodies, antisense oligonucleotides (ASO), and gene editing techniques. In animal and human studies, these substances (apoCIII and ANGPTL3 inhibitors) reduced the levels of apoCII and ANGPTL2 in plasma by approximately 70% and 80%, respectively. Moreover, studies have shown that antisense oligonucleotides targeting apoCIII can significantly reduce triglyceride levels even in FCS patients, making it the first drug currently available for these patients (Gugliucci).

The current research results indicate that TG and TRLs may become new potential targets for AS detection and treatment besides LDL-C. Further comprehensive understanding of potential pathological biology and related clinical research are crucial for the development of new biomarkers and therapeutic drugs. At present, there is no reliable technology to directly measure serum TRLs and TRLs residues in clinical practice, which poses a challenge in accurately assessing the risk and prognosis of cardiovascular diseases such as coronary heart disease and metabolic diseases such as diabetes. It is urgent to develop new, standardized and practical residue detection methods. Due to sufficient reduction of LDL-C, patients with cardiac metabolic abnormalities are still at high risk of disease recurrence. Therefore, the development of novel targeted drugs related to TRLs has important clinical and social significance.
